# Melittin Nanoparticles Mitigate Glyphosate-Induced Nephrotoxicity via Cytokine Modulation and Bax/Nrf2 Pathways

**DOI:** 10.3390/biomedicines13112607

**Published:** 2025-10-24

**Authors:** Amany M. Hamed, Zeyad Elsayed Eldeeb Mohana, Azza M. A. Abouelella, Wafaa A. Abdellah, Dalia A. Elbahy, Noha A. R. Fouda, Dina M. Monir, Safaa S. Soliman, Ahmed Mohamed Mahmoud Abdelfattah Elkassas, Elsayed Eldeeb Mehana Hamouda, Hany M. R. Abdel-Latif, Ahmed R. H. Ahmed, Nadia S. Mahrous

**Affiliations:** 1 Chemistry Department, Faculty of Science, Sohag University, Sohag 82524, Egypt; 2 College of Medicine, Alexandria University, Alexandria 26571, Egypt; 3 Department of Clinical Pharmacology, Faculty of Medicine, Sohag University, Sohag 82524, Egypt; 4 Department of Human Anatomy and Embryology, Sohag University, Sohag 82524, Egypt; nohafouda544@yahoo.com; 5 Physiology Department, Faculty of Medicine, Sohag University, Sohag 82524, Egypt; 6 Department of Zoology, Faculty of Science, Minia University, Minia 61519, Egypt; 7 Department of Pathology, College of Veterinary Medicine, Alexandria University, Alexandria 26571, Egypt; elsayedhamouda@gmail.com; 8 Department of Poultry and Fish Diseases, Faculty of Veterinary Medicine, Alexandria University, Alexandria 26571, Egypt; 9 Department of Pathology, Faculty of Medicine, Sohag University, Sohag 82524, Egypt; 10 Department of Zoology, Faculty of Science, South Valley University, Qena 83523, Egypt

**Keywords:** glyphosate, nephrotoxicity, melittin, oxidative stress, Nrf2 signaling, Bax expression, inflammation, apoptosis

## Abstract

**Background/Objectives:** Glyphosate-based herbicides (GBHs) are widely used agrochemicals implicated in nephrotoxicity through mechanisms involving oxidative stress, inflammation, and tissue remodeling. Natural peptides such as melittin possess potent anti-inflammatory and antioxidant properties; however, their therapeutic use is limited by instability and toxicity. Nanotechnology-based encapsulation presents a promising approach to overcoming these challenges. Objective: This study aimed to evaluate the protective effects of melittin-loaded chitosan–TPP nanoparticles (MEL-NPs) against glyphosate-induced nephrotoxicity in rats, with emphasis on oxidative, inflammatory, and apoptotic pathways. **Methods:** Female Wistar rats were divided into four groups: control, glyphosate (5 mg/kg/day, 25 days), glyphosate + free melittin, and glyphosate + MEL-NPs (40 µg/kg, orally, 3 times/week). Renal function biomarkers, oxidative stress parameters (MDA, GSH, SOD, CAT, NO), cytokines (TNF-α, IL-6), and serum protein/iron indices were assessed. Western blotting (Nrf2, NGAL), histopathology (H&E), and immunohistochemistry (Bax) were performed. Nanoparticles were characterized by TEM, FTIR, and UV–Vis spectroscopy. **Results:** Glyphosate exposure caused renal dysfunction, including elevated plasma urea and creatinine levels, and reduced creatinine clearance, indicating impaired glomerular filtration efficiency, oxidative stress (↑increased MDA, NO; ↓decreased GSH, SOD), and upregulation of pro-inflammatory cytokines. Histology revealed tubular degeneration and inflammatory infiltration, while NGAL and Bax were strongly induced. Nrf2 expression was elevated as a compensatory response. Free melittin partially ameliorated these alterations, whereas MEL-NPs provided superior protection, restoring renal function, normalizing oxidative balance, reducing NGAL and Bax expression, and preserving renal histoarchitecture. **Conclusions:** Melittin nanoparticles confer robust renoprotection against glyphosate-induced nephrotoxicity in rats by modulating oxidative stress, suppressing inflammation, and regulating Nrf2/Bax signaling. These findings highlight nano-melittin as a promising therapeutic platform for managing herbicide-related renal disorders.

## 1. Introduction

Glyphosate-based herbicides (GBHs), including Roundup^®^, are among the most widely used agrochemicals globally. Their pervasive environmental presence has raised significant concerns over human and animal exposure, especially regarding renal toxicity. In murine and rat models, GBH exposure has been linked to glomerular damage, tubular epithelial degeneration, inflammation, and necrosis, often mediated through oxidative stress mechanisms—characterized by overproduction of reactive oxygen species (ROS), elevated malondialdehyde (MDA) levels (a marker of lipid peroxidation), and depletion of antioxidant defenses such as glutathione (GSH) and antioxidant enzymes [1]. Specifically, Rihani et al. (2025) demonstrated that glyphosate undermines mitochondrial respiration in renal tubular cells, directly elevating oxidative injury in both cortex and medulla [2].

Melittin, the principal bioactive peptide of bee venom, is renowned for its anti-inflammatory, anti-apoptotic, and antioxidant properties. In murine sepsis-induced acute kidney injury (AKI), melittin significantly reduced renal cytokine expression (e.g., TNF-α, IL-6), suppressed NF-κB activation, and decreased lipid peroxidation markers such as 4-HNE and MDA, while upregulating Nrf2-HO-1 and other antioxidant pathways [3]. Furthermore, melittin enhanced GPX4 expression by stimulating Nrf2 nuclear translocation, thereby mitigating ferroptosis and preserving renal architecture in septic models [4]. In a chronic kidney failure model (5/6 nephrectomy), melittin also alleviated podocyte injury by inducing autophagy and improving markers such as podocin and nephrin, suggesting regulatory effects on mTOR signaling and autophagic flux [5].

Despite this promising evidence, melittin’s potential against herbicide-induced nephrotoxicity remains unexplored, particularly in the context of glyphosate exposure. Moreover, while nanoparticle-based formulations (e.g., propolis nanoparticles) have shown enhanced mitigation of glyphosate-induced oxidative stress in aquatic species and improved antioxidant status compared to native extracts [6], nanoformulations of melittin have not yet been evaluated in herbicide-induced renal injury models.

Therefore, the present study aims to explore, for the first time, the potential of melittin and its nanoformulation in mitigating glyphosate-induced nephrotoxicity in rats through modulation of oxidative stress, inflammation, ferroptosis, and autophagy pathways. The novelty of this work lies in its dual strategy: first, evaluating melittin, a bioactive peptide derived from bee venom, as a therapeutic agent against herbicide-induced renal injury, an area that remains largely uninvestigated; and second, incorporating nanotechnology to enhance its bioavailability, improve renal targeting, and reduce systemic toxicity. By integrating melittin’s multifaceted reno-protective mechanisms with nano-encapsulation, this study offers a novel therapeutic approach to counteract glyphosate-triggered oxidative and cellular damage, paving the way for future development of peptide-based nano-therapeutics.

## 2. Materials and Methods

### 2.1. Chemicals and Reagents

Glyphosate and melittin were obtained from Sigma-Aldrich (St. Louis, MO, USA) and stored in their original containers at room temperature in a cool, dry, and well-ventilated area throughout the study. Chitosan derived from shrimp shells (*Pandalus borealis*), with a deacetylation degree (DD) of 93% and low molecular weight, was purchased from Oxford Laboratory Chemicals (Maharashtra, India). Sigma-Aldrich also supplied sodium tripolyphosphate (STPP).

Serum ferritin (Cat. No. F048SC) and total iron-binding capacity (TIBC) (Cat. No. T181SC) were quantified using ELISA kits (HCB, Elixir Canada Medicine Company Ltd., Vancouver, BC, Canada) according to the manufacturer’s instructions. Serum iron concentrations were determined using a commercial kit (Cat. No. A039-1-1; Nanjing Jiancheng Bioengineering Institute, Jiangsu, China).

Oxidative stress markers, including catalase (CAT; Cat. No. A007-1-1), glutathione (GSH; Cat. No. A006-2-1), superoxide dismutase (SOD; Cat. No. A001-3), and malondialdehyde (MDA; Cat. No. A003-1) were assessed using assay kits from Nanjing Jiancheng Bio-Technology Co., Ltd. (Nanjing, China). Nitric oxide (NO) levels were measured using a commercial kit (Product No. NB 98; Biomax Co., Ltd., Guri-si, Republic of Korea) according to the provided protocols. Pro-inflammatory cytokines, tumor necrosis factor-alpha (TNF-α; Cat. No. RTA00-1) and interleukin-6 (IL-6; Cat. No. R6000B), were analyzed using ELISA kits from R&D Systems (Shanghai, China).

Renal function markers, including plasma urea and creatinine, were determined spectrophotometrically using standard diagnostic kits (Labtest Diagnostica, Lagoa Santa, Brazil). Serum total protein was measured using a kit (Cat. No. 310005; Spectrum Diagnostics, Cairo, Egypt), while serum albumin was assessed using a biochemical kit (Cat. No. AB 1010; Bio-Diagnostic Co., Cairo, Egypt), following the manufacturer’s protocols.

The optical density (OD) of each sample was recorded at 450 nm using a Multiskan Mk3 Microplate Reader (Thermo Scientific, Waltham, MA, USA). Analyte concentrations were calculated by interpolation from standard calibration curves.

### 2.2. Preparation of Nano-Formulated Melittin

Melittin-loaded chitosan nanoparticles (MEL-NPs) were prepared using a modified ionic gelation method based on previously reported protocols by El-Sawi et al. [7]. The nano formulation process was carried out at the Faculty of Science, Sohag University, Egypt. Briefly, melittin was dissolved in deionized water under constant stirring. Chitosan was separately dissolved in 1% (*v*/*v*) glacial acetic acid at room temperature and diluted with distilled water. The chitosan solution was then added dropwise to the melittin solution under continuous magnetic stirring at 500 rpm (Thermolyne, Thermo Scientific, USA). To initiate nanoparticle formation, a sodium tripolyphosphate (TPP) solution was added dropwise (one drop every three seconds) to the chitosan–melittin mixture using a burette, while maintaining stirring at 500 rpm. The mixture was stirred for an additional 30 min to ensure complete cross-linking and formation of stable melittin-loaded nanoparticles. The resulting MEL-NPs were collected by ultracentrifugation (Hanil Micro 17TR centrifuge—HE5, Hanil Scientific Inc., Gimpo-si, Republic of Korea) at 17,000 rpm for 30 min at 4 °C. The pellets were then freeze-dried (lyophilized) and stored at 4 °C for further analysis. The stability of the MEL-NPs was monitored over five days by assessing physical parameters, including color, turbidity, and sedimentation. Characterization of the nanoparticles included ultraviolet–visible (UV–Vis) absorption spectrum, Fourier-transform infrared spectroscopy (FTIR), drug loading capacity (DLC), entrapment efficiency (EE), average particle size, Size distribution, and transmission electron microscopy (TEM) imaging.

### 2.3. Evaluation and Characterization of Melittin Nanoparticles

#### 2.3.1. Particle Size, Distribution, and Morphology

The surface morphology, particle size, and size distribution of the melittin-loaded chitosan nanoparticles (MEL-NPs) were evaluated using transmission electron microscopy (TEM) (JEOL JEM 100 CXII, 100 kV, JEOL, Tokyo, Japan). This technique allowed the visualization of the nanoparticles’ shape and aggregation state, as well as estimation of the average particle diameter. The sample intended for transmission electron microscopy (TEM) analysis was prepared and examined following the procedure described in [8].

#### 2.3.2. Determination of Free Melittin Content by UV–Vis Spectroscopy

The ultraviolet–visible (UV–Vis) absorption spectrum of the melittin-loaded nanoparticles was recorded to quantify the unencapsulated (free) melittin remaining in the supernatant after nanoparticle preparation. Measurements were performed at a wavelength of 280 nm, which corresponds to the characteristic absorbance of melittin due to the presence of aromatic amino acids such as tryptophan and tyrosine. Water was used as a reference blank in the quartz cuvette. The obtained absorbance values were compared with a pre-established calibration curve of pure melittin to calculate the concentration of free drug in the supernatant [9].

#### 2.3.3. Fourier Transform Infrared Spectroscopy (FTIR)

Fourier Transform Infrared (FTIR) spectroscopy was conducted to assess the molecular interactions between chitosan and melittin, and to identify characteristic functional groups involved in nanoparticle formation [10]. FTIR spectra were recorded using an ATR-FTIR spectrometer (Alpha Bruker Platinum, 1-211-6353, Bruker, Billerica, MA, USA). Specific absorption bands corresponding to chemical bonds within the melittin–chitosan matrix were analyzed to confirm encapsulation and structural integrity.

#### 2.3.4. Determination of Encapsulation Efficiency (EE) and Drug Loading Capacity (DLC)

To determine the encapsulation efficiency (EE%) and drug loading capacity (DLC%) of melittin within the chitosan nanoparticles, the nanoparticle suspension was subjected to ultracentrifugation at 17,000 rpm for 30 min at 4 °C (Hanil Micro 17TR centrifuge—HE5). The concentration of unencapsulated (free) melittin in the supernatant was quantified using a UV–visible spectrophotometer at a specific wavelength characteristic for melittin. The EE% and DLC% were calculated using the following equations according to Cevher et al. [11]:

Encapsulation efficiency (%) = [(T − F)/T] × 100

Drug loading capacity (%) = [(T − F)/W] × 100

where T is the total amount of melittin added to the chitosan solution (mg), F is the free (non-encapsulated) melittin measured in the supernatant (mg), and W is the total weight of dried nanoparticles (mg)

### 2.4. Animals and Ethical Statement

Twenty-four healthy adult male Wistar rats weighing 180 ± 10 g were used in this study. All animal procedures and experimental protocols were reviewed and approved by the Institutional Animal Care and Use Committee (IACUC) at the Faculty of Veterinary Medicine, Alexandria University, Alexandria, Egypt, under approval number Au013250820250372. The rats were housed under standard laboratory conditions with a controlled temperature of 25 ± 2 °C, relative humidity of 65 ± 10%, 12 h light/dark cycle, and 11–13 air exchanges per hour. Animals were acclimatized for two weeks before experimentation and had free access to standard rodent chow and water ad libitum. All efforts were made to minimize animal discomfort and suffering throughout the study.

Female Wistar rats were selected for the present study in view of several prior reports indicating that renal injury due to oxidative and inflammatory toxic insults is as severe—and in some contexts even more pronounced—in females compared to males. For example, a study using a high-cholesterol diet found greater nephrotoxic damage in female Wistar rats than males [12]. Similarly, comparative studies of cadmium toxicity showed more marked biochemical and histopathological alterations in female rat kidneys than in male counterparts [13]. Moreover, studies of pesticide exposure in female rats (e.g., low-dose deltamethrin) demonstrated significant oxidative stress and kidney dysfunction in females [14]. These data suggest that females provide a sensitive model for detecting renal oxidative and inflammatory damage, allowing for clearer assessment of protective interventions.

### 2.5. Experimental Design

Twenty-four adult rats were randomly allocated into four experimental groups (*n* = 6 per group) as follows:

Group I (Control): Received phosphate-buffered saline (PBS) by oral gavage, serving as the vehicle control.

Group II (Glyphosate): Received glyphosate (5 mg/kg/day) via oral gavage for 25 consecutive days.

Group III (Glyphosate + Free Melittin): Received glyphosate as in Group II, followed by free melittin treatment (40 µg/kg orally, three times/week for one month).

Group IV (Glyphosate + Melittin Nanoparticles): Received glyphosate as in Group II, followed by treatment with melittin-loaded nanoparticles (40 µg/kg orally, three times/week for one month).

At the end of the treatment period, the animals were housed individually in metabolic cages for 24 h urine collection. The dose of glyphosate previously used by Nacano et al. [15]. While the dose of melittin and its nanoformulation was chosen based on our previous experiments, as described by El-Sawi et al. [7].

Melittin and melittin-loaded nanoparticles (MEL-NPs) were administered three times per week rather than daily to minimize potential cumulative toxicity and local irritation associated with repeated peptide injections, as reported in previous studies. Indeed, it has been shown that a controlled and prolonged release of melittin from nanocomplexes significantly reduces its acute toxicity in vivo [16]. This dosing frequency was sufficient to maintain therapeutic plasma concentrations of melittin while ensuring animal safety.

### 2.6. Sample Collection and Tissue Processing

At the end of the experimental period, blood samples were collected from the heart. Serum was separated within one hour of collection by centrifugation and stored at −20 °C until analysis. The kidneys were carefully dissected and rinsed with sterile saline. One kidney from each animal was minced and homogenized in ice-cold Tris-HCl buffer (pH 7.4), followed by centrifugation at 3000× *g* for 10 min. The resulting supernatant was collected and used for biochemical analyses, including enzymatic and non-enzymatic markers of oxidative stress. The second portion of the kidney tissue was fixed in 10% neutral-buffered formalin for histopathological examination.

### 2.7. Kidney Weight Measurement

At the time of sacrifice, both kidneys were carefully excised, rinsed with cold saline to remove blood residues, and blotted dry using filter paper. Absolute kidney weight in all groups was measured using an AD-5000 electronic balance (Marte Científica Ltd.a, São Paulo, Brazil), and the values were expressed in grams.

### 2.8. Analysis of Kidney Biomarkers

Plasma levels of urea and creatinine were quantitatively determined using standard colorimetric methods and commercially available diagnostic kits (Labtest Diagnostica, Lagoa Santa, Brazil), following the manufacturer’s instructions. The results were expressed in mg/mL. Creatinine clearance, an indicator of renal filtration function, was calculated using the following formula: Creatinine clearance (mL/min) = ((Urine creatinine concentration × Urine volume)/(Plasma creatinine concentration × 1440)), where 1440 represents the number of minutes in 24 h. Where 1440 represents the number of minutes in 24 h.

Serum globulin levels were subsequently calculated by subtracting the albumin concentration from the total protein: Globulin (g/dL) = Total Protein (g/dL) − Albumin (g/dL)

### 2.9. Western Blot

#### 2.9.1. Protein Extraction and Purification

Total proteins were extracted from kidney tissue using the TriFast™ reagent (Peqlab, VWR, Darmstadt, Germany), which allows simultaneous isolation of RNA, DNA, and protein according to the manufacturer’s instructions. Briefly, 50–100 mg of tissue was homogenized in 1 mL TriFast using a glass–Teflon homogenizer. Following chloroform addition and centrifugation (12,000× *g*, 5 min, 4 °C), the mixture separated into aqueous (RNA), interphase (DNA), and organic (protein) phases. The protein fraction was precipitated with isopropanol, washed three times with 0.3 M guanidinium hydrochloride in 95% ethanol, and finally solubilized in 1% Sodium Dodecyl Sulfate (SDS). Protein concentration was determined by the Bradford assay using bovine serum albumin (BSA) as a standard [17].

#### 2.9.2. Sodium Dodecyl Sulfate-Polyacrylamide Gel Electrophoresis (SDS-PAGE) and Western Blotting

Equal amounts of protein (30 μg) were separated by 10% SDS-PAGE and electro-transferred to Hybond™ nylon membranes (GE Healthcare, Chicago, IL, USA) [18]. Membranes were blocked with 5% non-fat dry milk in Tris-buffered saline with 0.1% Tween-20 (TBST) for 1 h at room temperature. For the immunoblotting analysis, primary antibodies were employed, including an NRF2 polyclonal antibody (Invitrogen, Carlsbad, CA, USA, PA5-27882, 1:1000 dilution; molecular weight ~97–100 kDa), an anti-NGAL antibody (Abcam, Cambridge, UK, ab63929; dilution 1:1000; molecular weight ~25 kDa), and an anti-β-actin antibody (Abcam, ab8226, 1:5000 dilution) as a loading control. To visualize the bound primary antibodies, a horseradish peroxidase (HRP)-conjugated goat anti-rabbit IgG secondary antibody was applied at a working concentration of 0.1–0.5 µg/mL. After overnight incubation at 4 °C, membranes were washed in TBST, and signals were detected using enhanced chemiluminescence (ECL). Band intensities were then quantified by densitometric analysis using ImageJ software (version 1.53t, National Institutes of Health, Bethesda, MD, USA).

All antibodies were handled according to the manufacturers’ protocols, and optimal dilutions were determined based on preliminary optimization experiments to achieve specific and reproducible signals.

### 2.10. Histopathological Studies

For the preparation of histological sections of different investigated groups, kidney tissue samples were fixed in 10% neutral buffered formalin for 24 to 36 h at room temperature. The fixed tissues were dehydrated at room temperature using ascending grades of ethyl alcohol, cleared in xylene, and embedded in paraffin at 70 °C. Paraffin blocks were sectioned at a thickness of 5 µm. The sections were deparaffinized in xylene, rehydrated through descending grades of ethanol, and rinsed in running tap water. Hematoxylin and eosin (H&E) staining was performed by incubating the sections in hematoxylin for 7 min at room temperature, followed by washing under running water and incubation in eosin for 2 min. The stained sections were rinsed again in running tap water, dehydrated through ascending grades of ethanol, cleared in xylene, and mounted using Dibutylphthalate Polystyrene Xylene (DPX) for microscopic examination.

### 2.11. Immunohistochemical Studies

Four-micrometer sections of formalin-fixed, paraffin-embedded kidney tissues were deparaffinized in xylene, rehydrated in descending grades of ethanol, and rinsed with running tap water. To block endogenous peroxidase activity, sections were incubated in 3% hydrogen peroxide (H_2_O_2_) for 10 min at room temperature. Antigen retrieval was performed by heating the sections in 0.01 mmol/L citrate buffer (pH 6.0) at 92 °C for 20 min. After cooling and washing with phosphate-buffered saline (PBS), the sections were incubated for 1 h at room temperature with the primary antibody: Anti-BAX rabbit monoclonal antibody (Clone SP47, Cat. No. ab81083, Abcam, Cambridge, UK). Following PBS washes, sections were incubated with an appropriate biotinylated secondary antibody (goat anti-rabbit for BAX), followed by incubation with streptavidin–biotin complex for 10 min. After washing with PBS, immunoreactivity was visualized using 3,3′-diaminobenzidine (DAB) as the chromogen for 15 min at room temperature (ScyTek, P.O. Box 3286, Logan, UT, USA). Nuclear counterstaining was performed using Harris’ hematoxylin for 2 min, followed by rapid washing with tap water. Sections were dehydrated in ascending alcohol concentrations, cleared in xylene, and mounted using DPX. The expression of BAX was assessed based on the percentage of positively stained cells and the intensity of staining. Examination and image capture were conducted using a binocular Olympus microscope (Model CX40 RF200, Olympus Optical Co., Ltd., Tokyo, Japan).

### 2.12. Statistical Analysis

Data were expressed as mean ± standard deviation (SD). Statistical comparisons between the experimental groups were performed using one-way analysis of variance (ANOVA), followed by Tukey’s post hoc test to determine significant differences between group means. * *p* < 0.05 was considered significant, ** *p* < 0.01 was considered highly significant, and *** *p* < 0.001 was considered extremely significant. An asterisk (*) denotes a significant difference compared to the control group. A hash symbol (#) denotes a significant difference compared to the glyphosate-treated group.

Pearson correlation coefficients were calculated to assess pairwise relationships among oxidative/nitrosative stress markers (MDA, NO, SOD, GSH, CAT), inflammatory cytokines (IL-6, TNF-α), and renal functional parameters (plasma urea, plasma creatinine, creatinine clearance). Correlations were two-tailed and considered significant at *p* < 0.05, with emphasis on associations at *p* < 0.01. Multivariate analyses included Principal Component Analysis to identify covariation patterns among biomarkers and multiple linear regression to evaluate independent predictors of renal dysfunction. All statistical analyses were performed using SPSS v27.

## 3. Results

### 3.1. Transmission Electron Microscopy (TEM) and Particle Size Distribution of Melittin Nanoparticles

**Figure 1:**
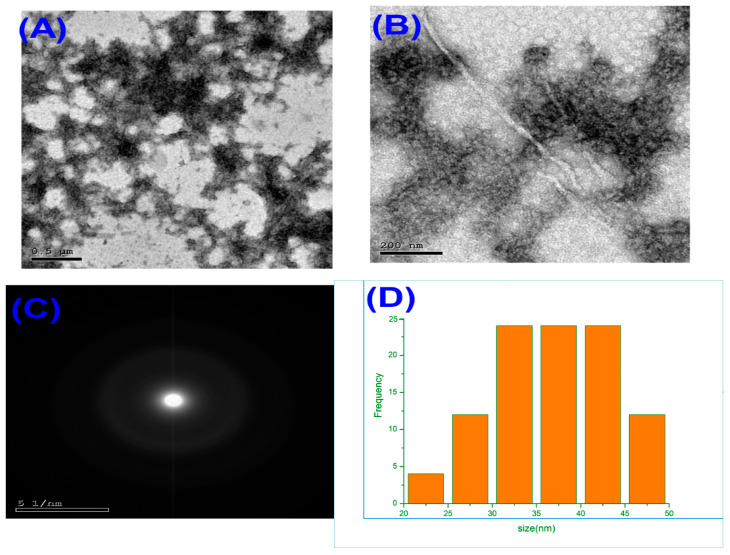
Transmission electron microscopy (TEM) images of melittin nanoparticles at different magnifications: (**A**) low-magnification image showing uniform distribution, (**B**) high-magnification image revealing spherical morphology, (**C**) selected area electron diffraction (SAED) pattern indicating amorphous structure, and (**D**) particle size distribution histogram showing an average diameter of ~36 nm with a narrow size range (20–50 nm).

Considering Transmission electron microscopy (TEM) images revealed that the synthesized melittin nanoparticles were uniformly distributed with a predominantly spherical morphology (Figure 1A,B). The nanoparticles appeared well-dispersed without significant aggregation, indicating the efficiency of the synthesis and stabilization process. Additional TEM micrographs at different magnifications are provided in the Electronic Supplementary Material (Figure S1), confirming consistent morphology and nanoscale particle size distribution. The average particle diameter, as determined from TEM measurements, ranged between approximately 25 and 45 nm, with most particles clustering around 35–40 nm. Selected area electron diffraction (SAED) patterns (Figure 1C) displayed diffuse concentric rings, confirming the amorphous nature of the melittin nanoparticles. The particle size distribution histogram (Figure 1D) demonstrated a narrow size distribution, with the majority of particles falling within the nanoscale range of 20–50 nm, and an average size of ~36 nm. This uniformity in size and spherical shape is advantageous for biological applications, as it promotes consistent cellular uptake and predictable pharmacokinetic behavior.

### 3.2. FTIR Spectral Characterization of Chitosan, Melittin, and Melittin Nanoparticles

**Figure 2:**
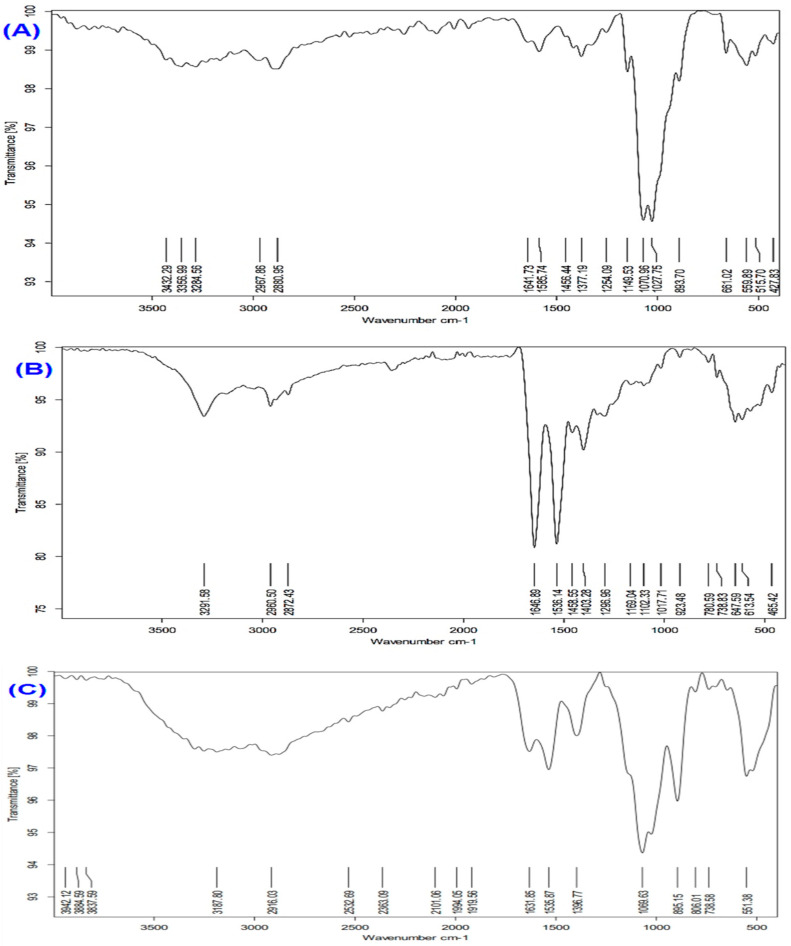
FTIR spectra of (**A**) chitosan, (**B**) melittin, and (**C**) melittin nanoparticles (Melittin-NPs). Characteristic peaks corresponding to functional groups in chitosan and melittin are observed, with notable shifts and changes in peak intensity in Melittin NPs, confirming the successful formation of nanoparticles through electrostatic interactions and hydrogen bonding between chitosan and melittin.

The FTIR spectra of chitosan, melittin, and melittin nanoparticles (MEL-NPs) are presented in Figure 2. The FTIR spectrum of chitosan (Figure 2A) exhibited broad absorption bands at 3432–3286 cm^−1^, corresponding to overlapping O–H and N–H stretching vibrations, confirming the presence of hydroxyl and amino groups. The absorption band at 1641 cm^−1^ was attributed to amide I (C=O stretching), while that at 1568 cm^−1^ corresponded to amide II (N–H bending). Additional peaks at 1150–1028 cm^−1^ were assigned to C–O–C stretching vibrations of the glycosidic bond in chitosan. The melittin spectrum (Figure 2B) revealed strong N–H stretching vibrations at 3291–2872 cm^−1^, an amide I band at 1646 cm^−1^, an amide II band at 1536 cm^−1^, and characteristic C–N and C–C stretching peaks between 1108–465 cm^−1^, consistent with its peptide backbone structure. For the melittin nanoparticles (MEL-NPs) (Figure 2C), significant spectral changes were observed. The amide I and amide II bands shifted from 1646 → 1631 cm^−1^ and 1536 → 1537 cm^−1^, respectively, accompanied by pronounced broadening of the O–H/N–H stretching region (3942–3187 cm^−1^). These shifts and broadening effects indicate hydrogen bonding and electrostatic interactions between the amino groups of chitosan and the peptide chains of melittin, confirming the successful encapsulation of melittin within the chitosan–TPP nanoparticle matrix.

### 3.3. Encapsulation Efficiency, Drug Loading Capacity, and UV–Vis Analysis

The UV–Vis spectrum of the nanoparticle preparation’s supernatant revealed a characteristic absorption peak at 280 nm, attributable to the aromatic amino acids (tryptophan and tyrosine) in melittin. The low absorbance intensity at this wavelength confirms that only a minimal portion of melittin remained unencapsulated, indicating successful loading into the chitosan-TPP nanocarrier. Quantitative assessment revealed an encapsulation efficiency (EE%) of 83%, demonstrating that the majority of melittin molecules were retained within the nanoparticles, thereby minimizing drug loss. In parallel, the drug loading capacity (DLC%), which reflects the ratio of the peptide mass to the total mass of nanoparticles, was 96%, underscoring the high payload potential of the developed nanoformulation.

### 3.4. Kidney Weight

**Figure 3:**
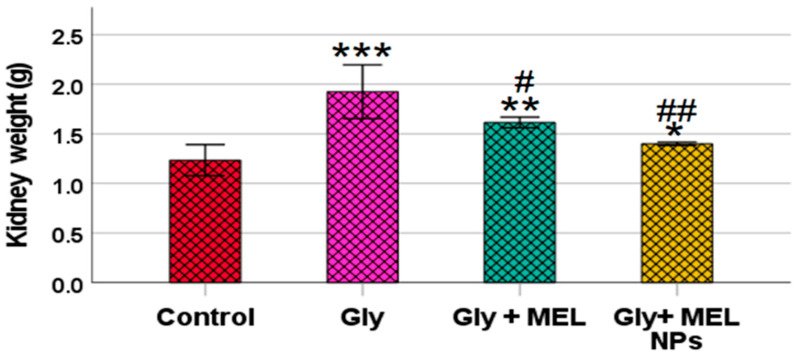
Effect of glyphosate, free melittin, and melittin nanoparticles on kidney weight in rats. Data are expressed as mean ± SD (*n* = 6). Gly: Glyphosate, MEL: melittin, MEL NPs: melittin nanoparticles. *** *p* < 0.001 vs. Control group; ** *p* < 0.01 vs. Control group; * *p* < 0.05 vs. Control group; ^##^ *p* < 0.01 vs. Glyphosate group and ^#^ *p* < 0.05 vs. Glyphosate group.

As illustrated in Figure 3, oral administration of glyphosate (5 mg/kg) for 25 days caused a significant increase in kidney weight compared to the control group (*** *p* < 0.001). However, treatment with free melittin significantly reduced kidney weight compared to the glyphosate group (## *p* < 0.01) and control group (** *p* < 0.01). Notably, treatment with melittin-loaded nanoparticles led to a more pronounced reduction, restoring kidney weight to near-control levels, with no statistically significant difference when compared to the control group (NS), but a highly significant difference compared to the glyphosate group (### *p* < 0.001).

### 3.5. Serum Iron Profile

**Figure 4:**
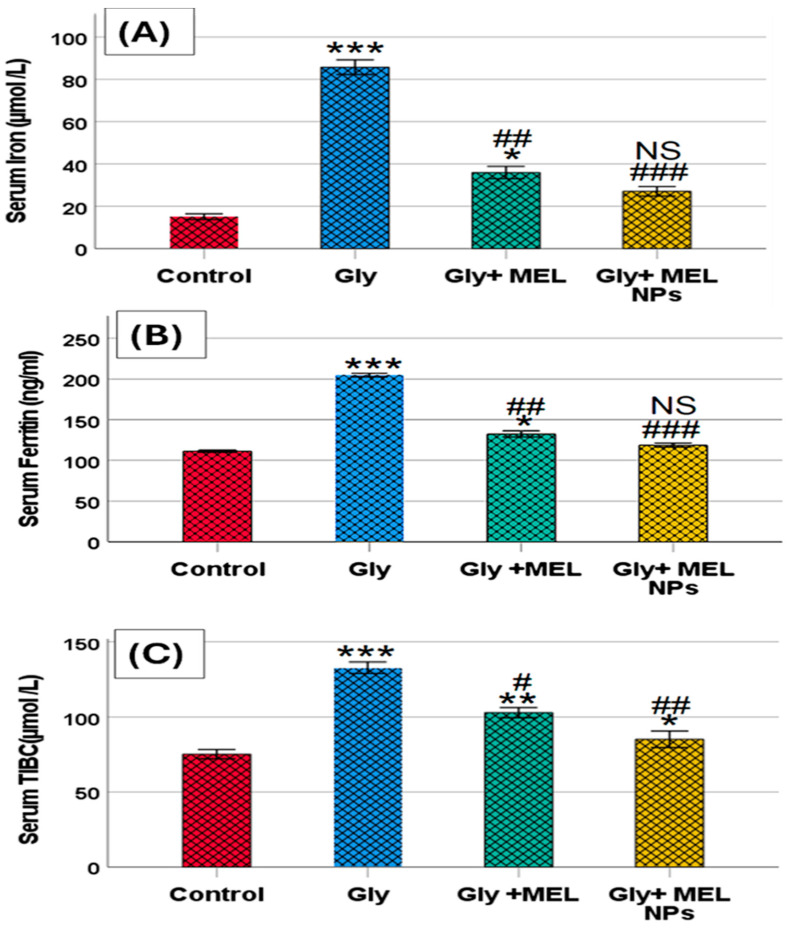
Effect of glyphosate, free melittin, and melittin nanoparticles on serum iron profile: (**A**) Serum iron (µmol/L), (**B**) Serum ferritin (ng/mL), and (**C**) Serum total iron-binding capacity (TIBC; µmol/L). Data are presented as mean ± SD (*n* = 6). Gly: Glyphosate, MEL: melittin, MEL NPs: melittin nanoparticles. Statistical significance: *** *p* < 0.001, ** *p* < 0.01 vs. Control group; * *p* < 0.05 vs. Control group; ^###^ *p* < 0.001 vs. Glyphosate group, ^##^ *p* < 0.01 vs. Glyphosate group; ^#^ *p* < 0.05 vs. Glyphosate group; and NS: non-significant vs. Control group.

As shown in Figure 4A–C, glyphosate administration resulted in a marked elevation in serum iron, ferritin, and total iron-binding capacity (TIBC) levels compared to the control group. Glyphosate-treated rats showed a significant increase in serum iron levels, which was effectively reduced upon treatment with free melittin and further reduced with melittin nanoparticles. Similarly, serum ferritin levels were elevated in the glyphosate group, while treatment with melittin and its nano formulation partially restored these levels toward normal values. Additionally, TIBC was significantly increased in the glyphosate group, and both melittin treatments were able to mitigate this elevation, with the nanoform showing greater efficacy.

### 3.6. Renal Function Biomarkers

**Figure 5:**
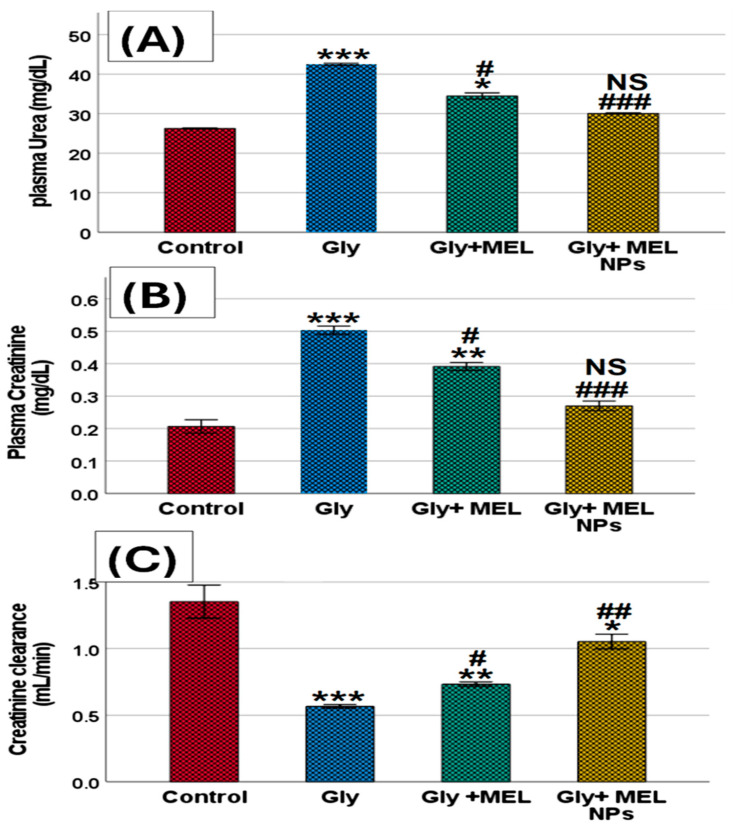
Effect of glyphosate, free melittin, and melittin nanoparticles on renal function biomarkers in rats: (**A**) Plasma urea (mg/dL), (**B**) Plasma creatinine (mg/dL), and (**C**) Creatinine clearance (mL/min). Data are expressed as mean ± SD (*n* = 6). Gly: Glyphosate, MEL: melittin, MEL NPs: melittin nanoparticles. Statistical analysis: *** *p* < 0.001, ** *p* < 0.01 vs. Control group; * *p* < 0.05 vs. Control group; ^###^ *p* < 0.001 vs. Glyphosate group, ^##^ *p* < 0.01 vs. Glyphosate group; ^#^ *p* < 0.05 vs. Glyphosate group; and NS: non-significant vs. Control group.

As shown in Figure 5A–C, glyphosate exposure induced profound alterations in renal functional indices. Plasma urea levels showed a significant elevation in the glyphosate-treated rats compared with the control group (*** *p* < 0.001), reflecting a marked disturbance in nitrogen metabolism. Administration of free melittin markedly reduced this elevation (* *p* < 0.05 vs. glyphosate, # *p* < 0.05), while treatment with melittin nanoparticles restored urea concentrations to values comparable to those of the control animals (NS vs. control, ### *p* < 0.001 vs. glyphosate).

A similar pattern was observed in plasma creatinine levels. Rats exposed to glyphosate exhibited a robust increase in creatinine compared with controls (*** *p* < 0.001), confirming impaired renal excretory function. Free melittin supplementation significantly attenuated this rise (** *p* < 0.01 vs. glyphosate, # *p* < 0.05), whereas melittin nanoparticles almost completely normalized creatinine values, showing no significant difference from the control group (NS vs. control, ### *p* < 0.001 vs. glyphosate).

In contrast, creatinine clearance was significantly decreased in the glyphosate group compared with controls (*** *p* < 0.001), indicating a loss of glomerular filtration efficiency. Treatment with free melittin led to a partial improvement (** *p* < 0.01 vs. glyphosate, # *p* < 0.05), while melittin nanoparticles produced a more pronounced effect, elevating clearance rates toward near-normal values (* *p* < 0.05 vs. glyphosate, ## *p* < 0.01).

Taken together, these results confirm that glyphosate disrupts renal function by elevating plasma urea and creatinine while reducing creatinine clearance, whereas melittin treatment, especially in nanoparticle form, provides significant renoprotection and preserves renal functional integrity.

### 3.7. Pro-Inflammatory Cytokines

**Figure 6:**
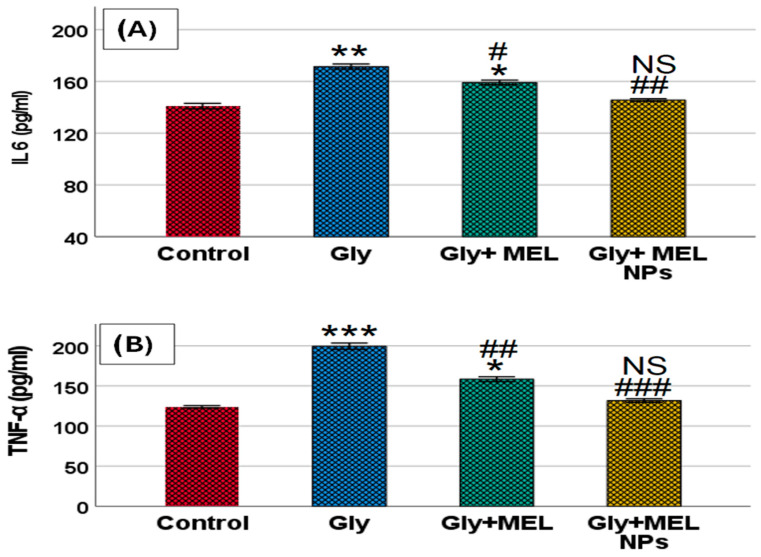
Effect of glyphosate, free melittin, and melittin nanoparticles on serum pro-inflammatory cytokine levels in rats. Bar graphs represent the serum concentrations of (**A**) interleukin-6 (IL-6) and (**B**) tumor necrosis factor-alpha (TNF-α) across experimental groups. Data are presented as mean ± SD (*n* = 6). Gly: Glyphosate, MEL: melittin, MEL NPs: melittin nanoparticles. Statistical significance: *** *p* < 0.001, ** *p* < 0.01 vs. Control group; * *p* < 0.05 vs. Control group; ^###^ *p* < 0.001 vs. Glyphosate group, ^##^ *p* < 0.01 vs. Glyphosate group; ^#^ *p* < 0.05 vs. Glyphosate group; and NS: non-significant vs. Control group.

As illustrated in Figure 6, glyphosate exposure significantly increased serum IL-6 and TNF-α compared with the control group. IL-6 (panel A): Rats treated with glyphosate exhibited a marked elevation in IL-6 (*p* < 0.01 vs. control). Free melittin treatment partially reduced IL-6 levels (* *p* < 0.05 vs. glyphosate, # *p* < 0.05), whereas melittin nanoparticles restored IL-6 toward baseline with non-significant differences compared to the control group (NS vs. control, ## *p* < 0.01 vs. glyphosate). Additionally, TNF-α (panel B): Glyphosate administration resulted in a robust rise in TNF-α (*** *p* < 0.001 vs. control). Free melittin significantly attenuated this increase (* *p* < 0.05 vs. glyphosate, ## *p* < 0.01), while melittin nanoparticles normalized TNF-α to near-control values (NS vs. control, ### *p* < 0.001 vs. glyphosate). Together, these findings demonstrate that glyphosate provokes a systemic pro-inflammatory response and that melittin, particularly in nanoparticle form, effectively mitigates cytokine overproduction.

### 3.8. Effect of Treatments on Oxidative Stress Biomarkers

Table 1 illustrates the impact of glyphosate, melittin, and melittin nanoparticles on oxidative stress markers and nitric oxide levels in rat tissues. Rats exposed to glyphosate exhibited a significant increase in CAT activity (875.14 ± 0.34 U/mg prot), MDA levels (4.28 ± 0.09 nmol/mg prot), and NO levels (29.45 ± 0.65 µmol/mL) compared to the control group (*** *p* < 0.001). Additionally, a marked reduction in antioxidant defenses was observed, including decreased SOD activity (85.84 ± 0.73 U/mg prot) and GSH content (3.37 ± 0.07 mg/g prot) (*** *p* < 0.001 vs. control), indicating a state of oxidative stress. Treatment with free melittin significantly mitigated glyphosate-induced oxidative damage, as evidenced by reduced MDA (3.29 ± 0.18 nmol/mg prot; ## *p* < 0.01), CAT (816.83 ± 0.75 U/mg prot; # *p* < 0.05), and NO levels (22.90 ± 0.49 µmol/mL; ## *p* < 0.01), along with an increase in GSH (5.15 ± 0.13 mg/g prot; *p* < 0.01) and SOD activity (89.38 ± 0.76 U/mg prot; # *p* < 0.05), when compared to the glyphosate group. Notably, treatment with melittin-loaded nanoparticles exhibited a more pronounced protective effect. The MDA and NO levels were further reduced (2.56 ± 0.16 and 19.38 ± 0.48, respectively; # *p* < 0.05 and ### *p* < 0.001 vs. glyphosate), while GSH and SOD levels were nearly restored to control values, with non-significant differences observed for CAT (794 ± 0.28 U/mg protein) and SOD (96.37 ± 0.47 U/mg protein) compared to the control. These findings highlight the superior antioxidative capacity of melittin nanoparticles in mitigating glyphosate-induced oxidative stress.

### 3.9. Correlation Between Oxidative/Antioxidant Status and Renal Function Parameters

Pearson correlation analysis revealed a strong, significant association between the pro-inflammatory cytokines (TNF-α and IL-6) and the oxidative stress indices, as shown in Table 2. Both TNF-α and IL-6 showed highly positive correlations with MDA (r = 0.965 and 0.967, respectively), NO (r = 0.973 and 0.967), and CAT activity (r = 0.982 and 0.971), and equally strong negative correlations with the antioxidant parameters SOD (r = −0.953 and −0.981) and GSH (r = −0.981 and −0.983). Moreover, elevated levels of TNF-α and IL-6 were strongly associated with higher plasma urea (r = 0.984 and 0.980) and creatinine (r = 0.972 and 0.984), whereas creatinine clearance exhibited significant negative correlations (r = −0.901 and −0.936, respectively). These findings indicate that enhanced oxidative and inflammatory responses were directly linked to the degree of renal dysfunction, while improvement of antioxidant status paralleled renal recovery in MEL-NP–treated rats.

### 3.10. Effect of Treatments on Total Protein, Albumin, and Globulin Levels

Table 3 shows the effect of glyphosate, melittin, and melittin-loaded nanoparticles on serum protein parameters. Rats exposed to glyphosate demonstrated a significant reduction in total protein (3.34 ± 0.17 mg/dL), albumin (2.10 ± 0.06 g/dL), and globulin (1.24 ± 0.21 g/dL) compared to the control group (*** *p* < 0.001), indicating impaired renal function and increased protein loss or catabolism. Treatment with free melittin partially restored protein levels, as reflected by a significant increase in total protein (5.84 ± 0.09 mg/dL; * *p* < 0.01 vs. control and ## *p* < 0.01 vs. glyphosate) and globulin (2.23 ± 0.11 g/dL; ** *p* < 0.01 vs. control and ## *p* < 0.01 vs. glyphosate), while albumin was restored to near-control levels with no significant difference (NS). In contrast, treatment with melittin nanoparticles produced a marked improvement. Levels of total protein (7.49 ± 0.42 mg/dL), albumin (3.71 ± 0.18 g/dL), and globulin (3.78 ± 0.34 g/dL) were restored to values comparable to the control group (NS), and significantly higher than those of the glyphosate group (### *p* < 0.001), highlighting the superior renoprotective effect of the nanoparticle formulation.

### 3.11. Nrf2 and NGAL Protein Expression

**Figure 7:**
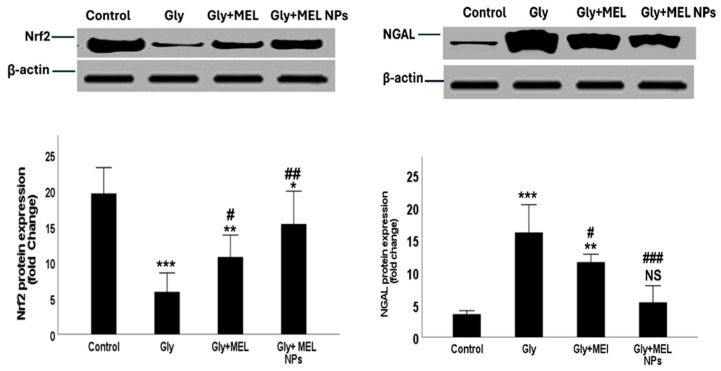
Representative Western blot images showing NRF2, NGAL, and β-actin protein expression in renal tissues of the four studied groups: Control, Glyphosate (Gly, toxic group), Melittin-treated group (MEL), and Melittin nanoparticle-treated group (MEL NPs). Corresponding densitometric analysis of band intensities (normalized to β-actin) is presented as histograms. Data are presented as mean ± SD (*n* = 3). Statistical significance; *** *p* < 0.001, ** *p* < 0.01, * *p* < 0.05 vs. Control group; ^###^ *p* < 0.001, ^##^ *p* < 0.01, ^#^ *p* < 0.05 vs. Glyphosate group; NS: non-significant vs. Control group.

Figure 7 shows Western blot analysis demonstrating distinct patterns of Nrf2 and NGAL protein expression among the four studied groups. In the Control group, basal Nrf2 expression was observed at low intensity, while NGAL expression was minimal, reflecting normal physiological conditions. In the Glyphosate group, Nrf2 protein expression showed a significant upregulation compared with the control, indicating activation of the antioxidant defense system in response to oxidative stress. At the same time, NGAL expression was markedly elevated, confirming glyphosate-induced renal injury. In the Melittin-treated group, Nrf2 expression exhibited partial restoration toward normal control levels, while NGAL expression was reduced compared with the glyphosate group, suggesting a moderate protective effect of free melittin. Interestingly, the Melittin nanoparticle-treated group showed the most pronounced normalization of Nrf2 expression, approaching control values, alongside a significant reduction in NGAL protein expression compared with both glyphosate and free melittin groups. This highlights the enhanced therapeutic efficacy of melittin in its nanoparticle formulation. β-actin bands confirmed equal protein loading across all lanes, ensuring reliable densitometric analysis l

### 3.12. Histological Results

**Figure 8:**
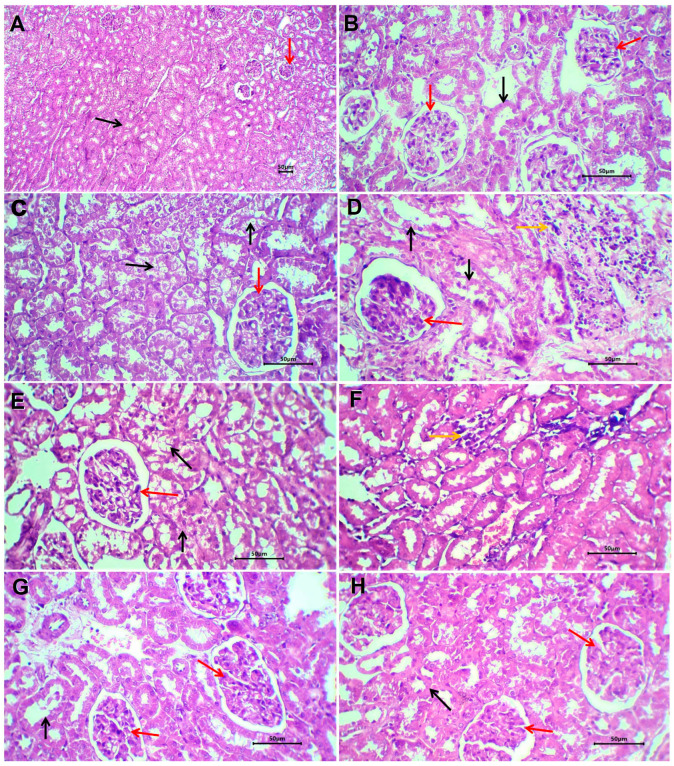
Sections of kidneys of non-treated rats (**A**,**B**), glyphosate-treated rats (**C**,**D**), glyphosate plus free melittin-treated rats (**E**,**F**), and glyphosate plus melittin nanoparticles-treated rats (**G**,**H**). Black arrows refer to renal tubules, red arrows refer to renal glomeruli, and yellow arrows refer to the inflammatory reaction. H&E-stained section; magnification is ×100 for (**A**) and ×40 for others.

The kidneys of rats that received PBS (negative control) showed no remarkable histological changes (Figure 8A,B). The renal cortex and medulla are identified. Renal glomeruli have rather normal Bowman’s space, and the glomeruli showed a normal capillary tuft. Renal tubules have a rather uniform size and shape and are lined by a single layer of cuboidal cells. Renal interstitial tissue showed mild congestion in a few scattered capillaries.

Renal tissue of rats treated with glyphosate showed patchy pathological changes (Figure 8C,D). The renal cortex and medulla are well-differentiated with no disruption. The glomeruli showed slight congestion of the glomerular capillary tuft with patent Bowman’s space. The lining epithelium of renal tubules showed atrophic changes in the form of cloudy swelling and of tubular epithelial lining with scattered cytoplasmic vacuoles. There is patchy disruption and shedding of the tubular lining epithelium. Renal interstitial tissue showed patchy infiltration by lymphocytes and a few neutrophils. There is focal congestion of interstitial blood vessels. No evidence of tissue necrosis or interstitial tissue hemorrhage could be seen.

Sections of renal tissue of rats treated with glyphosate and free melittin showed focal mild degeneration of renal tubules, residual mild inflammatory reaction, and scattered congested blood capillaries (Figure 8E,F). Renal tissue of rats treated with glyphosate plus melittin nanoparticles showed almost absent or minimal pathological changes (Figure 8G,H). There is focal mild cloudy swelling of the tubular lining epithelium with no shedded lining. Residual congestion of interstitial renal capillaries was observed with no encountered stromal inflammatory reaction.

### 3.13. Immunohistochemical Investigation of Bax Expression

**Figure 9:**
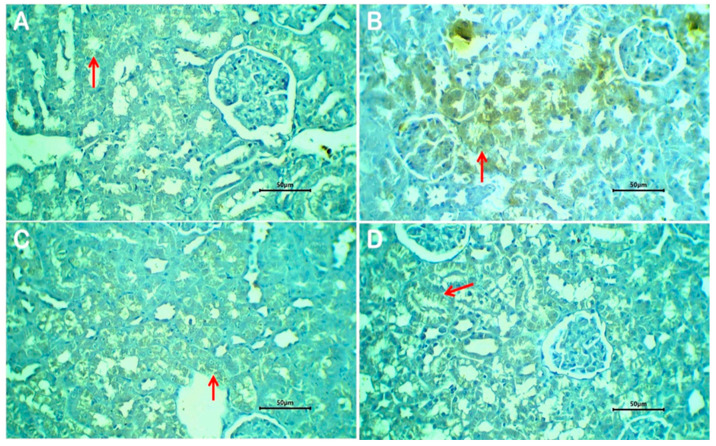
Expression of the BAX molecule in kidney tissue of non-treated rats (**A**), glyphosate-treated rats (**B**), glyphosate plus free melittin-treated rats (**C**), and glyphosate plus melittin nanoparticles-treated rats (**D**). Arrows refer to granular cytoplasmic immune staining of BAX in renal tubular epithelium. Immune-stained sections; magnification is ×40 for all.

Evaluation of the BAX protein, a pro-apoptotic molecule, was demonstrated as a granular cytoplasmic brown staining mainly in the epithelial cell lining of renal tubules. The expression was focal or patchy in all evaluated tissue samples. The immune reaction was faint in negative control rats (Figure 9A), strong in rats treated with glyphosate (Figure 9B), weak to moderate in rats treated with glyphosate/free melittin (Figure 9C), and in rats with glyphosate/melittin nanoparticles (Figure 9D).

## 4. Discussion

In this study, melittin nanoparticles (MEL-NPs) were successfully employed to investigate their potential renoprotective effects against glyphosate-induced nephrotoxicity. The findings demonstrated that glyphosate caused profound oxidative stress, an inflammatory response, and disruption of the protein profile in renal tissues, which were significantly alleviated by melittin, particularly in its nanoparticle formulation. The enhanced effect of melittin nanoparticles may be attributed to their unique physicochemical properties.

TEM analysis (Figure 1A,B) revealed that the synthesized melittin nanoparticles are uniformly spherical, with a tight size distribution within the nanoscale range (20–50 nm; average ~35–40 nm) and without significant aggregation. Spherical nanoparticles in this size range are advantageous for biomedical applications, facilitating enhanced tissue penetration and controlled biodistribution (e.g., renal retention) while minimizing recognition and clearance by the reticuloendothelial system (RES) [19]. The selected area electron diffraction (SAED) pattern (Figure 1C) displayed diffuse concentric rings, confirming that the nanoparticles possess amorphous organization—often associated with enhanced physical stability and slower release kinetics [20]. The narrow particle size distribution (Figure 1D) indicates controlled synthesis and reproducibility, critical for consistent biological interactions and pharmacokinetics. The morphological uniformity and absence of aggregation observed in TEM images (see Figure S1, Supplementary Material) further support the stability and homogeneity of MEL-NPs, which are critical parameters influencing their biological performance and renal targeting efficiency.

While X-ray diffraction (XRD) remains the gold standard for distinguishing crystalline from non-crystalline phases, in the present work, the amorphous character of MEL-NPs was inferred from TEM images lacking sharp lattice fringes and from SAED patterns that show diffuse halos rather than discrete spots. These observations align with literature reports for chitosan/TPP nanoparticle systems, where ionotropic crosslinking disrupts the native crystalline domains of chitosan, yielding particles with an amorphous-like XRD pattern [21]. As a limitation, actual XRD measurement was not conducted in this study due to limited sample availability, but it will be included in future work to quantitatively corroborate the amorphous structure.

The FTIR findings further support the successful formation of melittin-loaded chitosan nanoparticles. The observed red shift of the amide I band (1646 → 1631 cm^−1^) and the slight shift of the amide II band (1536 → 1537 cm^−1^), together with the broadening of the O–H/N–H stretching region (3942–3187 cm^−1^), indicate strong hydrogen bonding and electrostatic interactions between the amino groups of chitosan and the peptide moieties of melittin. These spectral modifications suggest that melittin molecules were efficiently incorporated within the chitosan–TPP polymeric matrix through ionic crosslinking and hydrogen-bond stabilization rather than simple physical adsorption. Similar spectral shifts have been reported in other peptide-loaded chitosan nanocarriers, confirming that such interactions contribute to enhanced peptide stability, controlled release, and reduced hemolytic activity [20]. Thus, the FTIR data corroborate the physicochemical evidence that melittin encapsulation was successfully achieved, resulting in a stable and biocompatible nanoparticulate formulation with enhanced functional properties.

The high encapsulation efficiency (83%) and drug loading capacity (96%) obtained for melittin-loaded chitosan–TPP nanoparticles strongly indicate the suitability of this nanocarrier system for peptide delivery. The relatively low UV–Vis absorbance at 280 nm, corresponding to aromatic amino acids in melittin, confirmed that only a small fraction of free peptide remained unencapsulated, supporting the efficiency of the ionic gelation method in entrapping melittin. These findings are in close agreement with recent literature on chitosan-based nanoparticle formulations. For instance, a comprehensive review by Obeidat et al. (2022) [22] demonstrated that drug solubility and formulation conditions critically influence entrapment efficiency and particle size in chitosan–TPP systems, underscoring the importance of optimizing these parameters for achieving a high payload. Similarly, a 2025 study in Polymers reported that chitosan nanoparticles consistently achieve encapsulation efficiencies exceeding 80–90%, attributed to their biocompatibility, high surface area, and porous structure, which facilitate both electrostatic and physical interactions with the drug [23]. These structural characteristics enhance nanoparticle stability and drug retention, aligning well with our observations. Collectively, the present results highlight that optimized chitosan–TPP nanoparticles represent a promising platform for the efficient encapsulation and sustained delivery of melittin, providing both high drug retention and potential therapeutic stability.

In the present study, glyphosate exposure resulted in a significant elevation of plasma urea and creatinine, accompanied by a reduction in creatinine clearance, reflecting a clear impairment of renal function. These findings are in agreement with previous reports demonstrating that glyphosate-based herbicides (GBHs) exert nephrotoxic effects through oxidative stress, mitochondrial dysfunction, and disruption of tubular reabsorption, ultimately leading to impaired nitrogen metabolism and filtration efficiency [1]. Similar patterns of elevated serum urea and creatinine have been observed in rodent models following glyphosate administration, confirming that these parameters are sensitive indicators of chemical-induced renal injury [24].

The decline in creatinine clearance in glyphosate-exposed rats suggests glomerular dysfunction, which may represent an early stage of renal insufficiency. Epidemiological studies have also highlighted glyphosate exposure as a risk factor for renal injury, with alterations in estimated glomerular filtration rate (eGFR) and biomarkers of tubular damage such as KIM-1 and NGAL being reported in human populations exposed to herbicides [25,26,27]. This concordance between animal and human studies strengthens the translational relevance of our findings.

Melittin administration ameliorated glyphosate-induced renal dysfunction, as evidenced by reductions in urea and creatinine levels and partial recovery of creatinine clearance. These results are consistent with reports that melittin exerts protective effects in renal injury models through its antioxidant and anti-inflammatory actions, mediated by suppression of NF-κB signaling and activation of the Nrf2/HO-1 pathway [3]. Importantly, melittin nanoparticles provided superior renoprotection, completely restoring biochemical indices toward normal values. The enhanced efficacy of the nanoparticle formulation can be attributed to improved peptide stability, reduced systemic degradation, and sustained renal delivery, as demonstrated in recent nanomedicine studies [28,29].

Taken together, these results suggest that glyphosate disrupts renal excretory function through oxidative and inflammatory mechanisms, while melittin, particularly in nanoformulated form, can effectively counteract these alterations. The findings underscore the therapeutic promise of melittin nanoparticles as a novel intervention against glyphosate-induced nephrotoxicity and potentially other oxidative and inflammatory kidney disorders.

Melittin has documented anti-inflammatory and antifibrotic properties. For instance, in the unilateral ureteral obstruction (UUO) model, melittin significantly inhibited inflammation and fibrosis by downregulating TGF-β1 and TNF-α–mediated pathways [30]. Additionally, melittin has been shown to induce protective autophagy in podocytes, mitigating chronic renal failure progression in the 5/6 nephrectomy rat model [5]. These renoprotective mechanisms likely contributed to the functional recovery observed in our glyphosate-injured rats, particularly when delivered via nanoparticles that enhance cellular uptake and reduce systemic clearance.

Our data confirms that subacute exposure to glyphosate perturbs redox homeostasis and ignites systemic inflammation, two converging pathways central to nephrotoxicity. Multiple recent studies show that glyphosate or glyphosate-based herbicides (GBHs) elevate lipid peroxidation (e.g., TBARS/MDA), depress antioxidant defenses, and trigger cytokine release, ultimately impairing renal structure and function. These effects are increasingly documented in rodent kidneys and align with our observation of higher MDA/NO and altered antioxidant enzyme activities in the glyphosate group [1,15].

In the glyphosate group, MDA and NO rose markedly, whereas SOD and GSH declined, canonical signatures of oxidative and nitrosative stress. Elevated CAT activity in our model likely represents a compensatory upregulation in response to excess H_2_O_2_, rather than true protection, a pattern observed after pesticide/GBH stress, where some antioxidant enzymes transiently increase while others decline. Mechanistically, glyphosate impairs mitochondrial respiration and increases ROS generation in renal tissue, while iNOS induction drives NO/peroxynitrite, compounding lipid peroxidation—consistent with our higher NO values. Treatment with melittin and especially melittin-NPs partially normalized these indices, indicating attenuation of ROS/RNS burden [2,31,32].

The strong correlations observed between TNF-α/IL-6 and oxidative stress markers (MDA, NO, CAT) indicate that glyphosate-induced renal injury is mechanistically associated with redox imbalance and inflammatory activation. The inverse relationship between cytokines and antioxidant enzymes (SOD, GSH) further supports the concept that depletion of endogenous antioxidants promotes the overproduction of reactive oxygen and nitrogen species, subsequently amplifying cytokine release and tissue damage. The significant positive correlations of TNF-α and IL-6 with plasma urea and creatinine, along with their negative association with creatinine clearance, emphasize the central role of oxidative-inflammatory stress in mediating glomerular dysfunction. Treatment with MEL-NPs effectively restored the antioxidant status and reduced cytokine levels, thereby breaking this pathogenic loop. These results are in line with reports showing that nano-delivery systems for melittin reduce its hemolytic toxicity and improve its stability and targeting (e.g., Wang et al., 2022; targeted counteracting of overactive macrophages via melittin-loaded lipid nanoparticles [33]).

In our rat model, glyphosate induced a pronounced systemic inflammatory response, as evidenced by marked elevations in serum IL-6 and TNF-α levels. This aligns with sub-chronic rodent studies demonstrating that glyphosate-based herbicides elicit pro-inflammatory cytokine release in peripheral tissues, including kidney and liver, along with increased oxidative stress, before overt histopathological renal damage manifests [34].

Free melittin administration attenuated these pro-inflammatory cytokine surges, while MEL-NPs) nearly normalized both IL-6 and TNF-α to control levels. This effect is consistent with reports showing that melittin suppresses NF-κB activation, reduces cytokine production, and enhances Nrf2-mediated antioxidant defenses in models of endotoxin- or sepsis-induced acute kidney injury [3,35,36]. In particular, Kim et al. (2021) demonstrated that melittin restores redox balance and inhibits inflammatory and apoptotic signaling in LPS-induced renal injury by activating Nrf2 and downregulating NF-κB pathways [3].

Furthermore, melittin has been shown to reduce TNF-α and IL-1β expression in chronic kidney injury models, such as unilateral ureteral obstruction, highlighting its broad anti-inflammatory efficacy across different etiologies of renal injury [30].

Taken together, our findings suggest that melittin, especially in nano form, confers renoprotection by dampening glyphosate-induced inflammatory cascades (IL-6, TNF-α), possibly through inhibition of NF-κB signaling and augmentation of the Nrf2 antioxidant axis. The superior efficacy of MEL-NPs over free melittin is likely due to improved peptide stability, bioavailability, and sustained renal delivery.

Several studies report that glyphosate exposure elevates classical kidney injury markers and disrupts renal histology via oxidative and inflammatory mechanisms. In our model (dose/duration as implemented), we observed functional alterations that do not fully mirror the classic azotemia pattern, suggesting possible early hemodynamic/tubular handling changes (e.g., hyperfiltration or osmoregulatory imbalance) rather than established filtration failure. This interpretation is consistent with reports that GBHs can alter renal hemodynamics and osmotic balance in subacute windows; nonetheless, histopathology and injury biomarkers (e.g., KIM-1, NGAL) would strengthen mechanistic assignment in future work [15,37].

The glyphosate group showed depressed total protein and globulins, with partial correction by melittin therapies. Systemically, GBHs provoke inflammation and oxidative stress that can impair protein synthesis and increase protein loss; renal barrier/tubular dysfunction may also contribute. The observed normalization (more pronounced with MEL-NPs) is consistent with the mitigation of oxidative/inflammatory stress and improved renal handling [38].

We observed higher serum iron and ferritin alongside increased TIBC in the glyphosate group. Ferritin rises as an acute-phase reactant with IL-6–driven hepcidin, while TIBC typically decreases in pure inflammation but increases with iron-restricted states. Glyphosate is a documented metal chelator that can bind divalent cations, including Fe; chelation could transiently distort iron distribution/transport and interact with inflammation to produce mixed patterns. The partial normalization by MEL-NPs likely reflects dampened inflammation (lower IL-6/TNF-α) and improved oxidative status, reducing ferritin up-regulation and restoring iron handling. Future work should include transferrin saturation (TSAT) and hepcidin to disentangle inflammatory versus chelation-driven effects [39,40,41,42].

Recent human analyses (NHANES) report that higher urinary glyphosate associates with lower serum iron and ferritin (trend *p* ≤ 0.01) and altered iron-binding metrics (UIBC/TIBC), suggesting disrupted iron availability and transport. Where our dataset shows shifts in ferritin/TIBC, the directionality mirrors these associations and is biologically plausible given glyphosate’s chelating propensity and oxidative milieu [43].

Mechanistically, glyphosate has been implicated in iron-handling stress (e.g., ferritinophagy/ferroptosis axes in related contexts), providing a framework for the ferritin/TIBC changes we observe [44].

Melittin exerts antioxidant-anti-inflammatory renoprotection by suppressing NF-κB/TNF-α signaling and engaging Nrf2/HO-1, as shown in recent kidney injury models (including aminoglycoside AKI) [4,7,45]. Our findings (trends toward lower cytokines and restored redox balance) echo these mechanisms. Nano-formulating melittin in chitosan–TPP carriers improves protease stability, reduces off-target hemolysis, and can enhance tissue exposure, rationalizing the greater biochemical recovery we observed with MEL-NPs. Notably, an independent study reported spherical CS-TPP melittin nanoparticles with high encapsulation and tangible anti-inflammatory/antioxidant benefits in vivo, supporting the translational plausibility of our approach. Preclinical work using bee-venom/melittin-loaded chitosan nanoparticles demonstrates robust antioxidant/anti-inflammatory effects and in vivo safety, paralleling our efficacy/safety signal for MEL-NPs [46,47,48].

Our findings show that glyphosate exposure triggered an upregulation of Nrf2 expression in the Glyphosate group, suggesting activation of the antioxidant defense pathway in response to oxidative stress. This aligns with current literature indicating that Nrf2 is a master regulator of cellular redox homeostasis and plays a protective role in kidney injury models, including acute and chronic damage [49,50]. Notably, the review by Ng et al. (2024) emphasizes that Nrf2 activation can alleviate kidney damage induced by nephrotoxic agents through antioxidant, anti-inflammatory, and anti-fibrotic actions [49].

The marked elevation of NGAL in the Glyphosate group reflects renal tubular damage, consistent with its status as a sensitive biomarker for acute kidney injury (AKI) [51]. The simultaneous increase in Nrf2 and NGAL supports the idea that oxidative stress and cellular injury are interlinked in glyphosate-induced nephrotoxicity.

Administration of free melittin partially restored Nrf2 expression toward control levels and reduced NGAL. This suggests that melittin possesses moderate nephroprotective properties, potentially mediated through Nrf2-modulated antioxidant responses. Recent work highlights melittin’s capacity to attenuate NOX4-mediated oxidative stress and activate Nrf2-associated pathways in renal tissue, offering protection against acute kidney injury. Additionally, melittin has been demonstrated to mitigate renal fibrosis and injury in obstruction models [3]. The Melittin nanoparticle-treated group showed the most pronounced normalization of Nrf2 and suppression of NGAL, suggesting a superior therapeutic effect compared to free melittin. This is consistent with advancements in nanotechnology aimed at mitigating melittin’s inherent cytotoxicity while preserving its pharmacological benefits. For example, polymer-based nanoparticles have been developed to reduce hemolysis and off-target toxicity of melittin, enabling safer delivery [52].

While Nrf2 activation is largely beneficial, its overactivation can be detrimental. Some studies caution that constitutive Nrf2 activation may exacerbate podocyte injury or contribute to adverse cardiovascular events in chronic kidney disease settings [53,54]. Therefore, the nanoparticle-mediated modulation seen here, bringing Nrf2 back toward normal, is likely more favorable than indefinite upregulation.

Our histopathological examination revealed that glyphosate exposure induced tubular degeneration, vacuolization, and inflammatory infiltration, consistent with early nephrotoxic damage. These morphological alterations were paralleled by a significant increase in NGAL expression, a sensitive biomarker of tubular injury, further confirming the presence of acute tubular stress. At the same time, glyphosate markedly upregulated expression, which likely reflects a compensatory antioxidant response to heightened oxidative stress within the renal parenchyma.

Treatment with free melittin attenuated tubular degeneration and reduced NGAL immunostaining, while partially normalizing Nrf2 expression. This suggests that melittin confers moderate protection by limiting tubular injury and rebalancing redox-sensitive transcriptional pathways. Importantly, melittin nanoparticles preserved normal renal histoarchitecture with minimal inflammatory infiltration, restored Nrf2 levels toward baseline, and strongly suppressed NGAL expression. The normalization of both structural features (histology) and molecular markers (Nrf2, NGAL) underscores the superior therapeutic efficacy of the nanoformulation.

These integrated findings agree with the previous report that glyphosate activates Nrf2 as a stress response while elevating NGAL as a damage signal [1]. Moreover, the study in rodent models of nephrotoxicity has shown that melittin mitigates tubular injury by suppressing NF-κB–driven inflammation and activating Nrf2/HO-1 pathways [3]. The convergence of histological recovery and molecular normalization in our study strongly supports the role of melittin nanoparticles as a renoprotective agent against glyphosate-induced kidney injury.

The enhanced renoprotective efficacy of MEL-NPs compared with free melittin can be quantitatively attributed to their physicochemical properties. The nanoparticles’ small, uniform size (~36 ± 5 nm; Figure 1D) facilitates passive renal accumulation via glomerular filtration and tubular retention, thereby increasing local drug exposure. Previous pharmacokinetic modeling suggests that nanoparticles smaller than 50 nm exhibit preferential renal deposition with extended tissue residence times, correlating with the improved recovery of renal function biomarkers (urea, creatinine clearance) observed in our study (Figure 5A–C). The amorphous nature of MEL-NPs, confirmed by SAED (Figure 1C), contributes to slower melittin release kinetics and sustained antioxidant effects, reflected by the greater normalization of MDA, GSH, and SOD values (Table 1). Furthermore, FTIR-detected hydrogen bonding and electrostatic interactions between the chitosan amino groups and melittin peptide chains (shifts in the amide I and II bands) likely stabilize the peptide against enzymatic degradation, ensuring its bioactive preservation in circulation. This stabilization correlates with the pronounced suppression of pro-inflammatory cytokines (IL-6, TNF-α; Figure 6) and Bax expression (Figure 9), together supporting a structure–activity relationship in which nanoscale encapsulation, amorphousity, and surface chemistry collectively underlie the superior renoprotective outcomes of MEL-NPs.

Although this study provides robust evidence for the renoprotective potential of melittin nanoparticles, some limitations should be noted. The modest sample size and relatively short exposure duration may have limited the detection of subtle changes. In addition, urinary biomarkers of renal injury such as KIM-1 and NGAL, which are currently regarded as gold-standard indicators of early tubular damage, were not assessed in this work. Incorporating these markers in future studies would provide more sensitive and translationally relevant insights. Moreover, pharmacokinetic and biodistribution studies of melittin nanoparticles, along with long-term safety evaluations, are warranted to support their clinical applicability.

## 5. Conclusions

This study demonstrated that glyphosate exposure induces profound renal toxicity, characterized by oxidative stress, upregulation of inflammatory cytokines, protein dysregulation, and histopathological deterioration, accompanied by increased immunostaining of pro-inflammatory (TNF-α, NF-κB) and pro-apoptotic (Bax) markers. Treatment with melittin-loaded chitosan–TPP nanoparticles provided significant protection by restoring antioxidant balance, activating Nrf2 signaling, suppressing NF-κB/TNF-α-mediated inflammation, and preserving renal architecture. The enhanced encapsulation efficiency and sustained release profile of the nanoformulation potentiated its therapeutic action compared to free melittin. Collectively, these findings highlight melittin nanoparticles as a promising nanotherapeutic strategy for mitigating glyphosate-induced renal injury and suggest their broader application against oxidative and inflammatory kidney disorders. Future studies with larger cohorts, extended treatment windows, and evaluation of biodistribution, pharmacokinetics, and long-term safety are warranted to support clinical translation.

## Figures and Tables

**Table 1:** Effect of glyphosate, melittin, and melittin nanoparticles on oxidative stress biomarkers in rat tissue homogenates.

Groups/Parameters	CAT (U/mg Protein)	MDA (nmol/mg Protein)	SOD (U/mg Protein)	GSH (mg/g Protein)	NO (Umol/mL)
Control	764.93 ± 0.76	1.86 ± 0.13	98. ± 0.71	7.82 ± 0.25	15.39± 0.29
Gly	875.14 ± 0.34 ***	4.28 ± 0.09 ***	85.84 ± 0.73 ***	3.37 ± 0.07 ***	29.45± 0.65 ***
Gly + MEL	816.83 ± 0.75 *^,#^	3.29 ± 0.18 **^,##^	89.38 ± 0.76 *^,##^	5.15 ± 0.13 **^,##^	22.90± 0.49 **^,##^
Gly + MEL NPs	794 ± 0.28 ^NS,##^	2.56 ± 0.16 *^,###^	96.37 ± 0.47 ^NS,###^	7.08 ± 0.15 ^NS,###^	19.38± 0.48 *^,###^

Data are expressed as mean ± SD (*n* = 6). *** *p* < 0.001, ** *p* < 0.01, * *p* < 0.05 compared to control group; ^###^ *p* < 0.001, ^##^ *p* < 0.01, ^#^ *p* < 0.05 compared to glyphosate group; NS: non-significant vs. control. CAT: catalase, MDA: malondialdehyde, SOD: superoxide dismutase, GSH: reduced glutathione, NO: nitric oxide. Gly: Glyphosate, MEL: melittin, MEL NPs: melittin nanoparticles.

**Table 2:** Pearson correlation coefficients (r) showing the relationship between inflammatory cytokines (TNF-α and IL-6) and oxidative stress/renal function parameters in glyphosate-exposed rats treated with MEL-NPs. (all subjects were grouped together).

Parameters	TNF-α (r)	IL-6 (r)	*p* Value
**MDA**	0.965	0.967	*p* < 0.001
**NO**	0.973	0.967	*p* < 0.001
**SOD**	−0.953	−0.981	*p* < 0.001
**GSH**	−0.981	−0.983	*p* < 0.001
**CAT**	0.982	0.971	*p* < 0.001
**Urea**	0.984	0.980	*p* < 0.001
**creatinine**	0.972	0.984	*p* < 0.001
**Creatinine clearance**	−0.901	−0.936	*p* < 0.001

Values represent Pearson correlation coefficients (r) between TNF-α, IL-6, and the indicated biochemical parameters. Positive correlations indicate direct relationships, while negative values indicate inverse associations. All correlations were statistically significant at *p* < 0.001 (two-tailed). **MDA:** malondialdehyde; **NO:** nitric oxide; **SOD:** superoxide dismutase; **GSH:** reduced glutathione; **CAT:** catalase; **TNF-α:** tumor necrosis factor alpha; **IL-6:** interleukin-6; **MEL-NPs:** melittin-loaded chitosan–TPP nanoparticles.

**Table 3:** Effect of glyphosate, melittin, and melittin nanoparticles on serum total protein, albumin, and globulin levels as renal function indicators in rats.

Groups/Parameters	Protein (g/dL)	Albumin (g/dL)	Globulin (g/dL)
Control	8.01 ± 0.18	3.58 ± 0.10	4.42 ± 0.26
Gly	3.34 ± 0.17 ***	2.10 ± 0.06 ***	1.24 ± 0.21 ***
Gly + MEL	5.84 ± 0.09 **^,##^	3.61 ± 0.08 ^NS,###^	2.23 ± 0.11 **^,##^
Gly + MEL NPs	7.49 ± 0.42 ^NS,###^	3.71 ± 0.18 ^NS,###^	3.78 ± 0.34 *^,###^

Data are presented as mean ± SD (*n* = 6). *** *p* < 0.001, ** *p* < 0.01, * *p* < 0.05 vs. Control group; ^###^ *p* < 0.001, ^##^ *p* < 0.01 vs. Glyphosate group; **NS:** non-significant vs. Control group. Globulin levels were calculated by subtracting albumin from total protein. **Gly:** Glyphosate, **MEL:** melittin, **MEL NPs:** melittin nanoparticles.

## Data Availability

All data generated or analyzed during this study are included in this published article.

## Supplementary Materials

The following supporting information can be downloaded at: https://www.mdpi.com/article/10.3390/biomedicines13112607/s1, Figure S1: TEM micrographs of melittin-loaded chitosan–TPP nanoparticles (MEL-NPs) at different magnifications. (A) Low-magnification image (0.5 µm scale) showing a uniform distribution of MEL-NPs with spherical to slightly irregular morphology and minimal aggregation. (B) Medium-magnification image (200 nm scale) illustrating distinct particle boundaries and homogeneous size distribution within the nanometer range. (C) High-magnification image (500 nm scale) revealing the amorphous internal texture of the nanoparticles with smooth, well-defined surfaces. Overall, the TEM images confirm good dispersion, nanoscale size uniformity, and the absence of significant crystalline domains, consistent with amorphous chitosan-based nanostructures.

## Abbreviations

GBHs	Glyphosate-based herbicides
ROS	Reactive oxygen species
MDA	Malondialdehyde
GSH	Reduced glutathione
GPX4	Glutathione peroxidase 4
mTOR	Mammalian target of rapamycin
TPP	Tripolyphosphate
EE%	Encapsulation efficiency
DLC%	Drug loading capacity
TEM	Transmission electron microscopy
FTIR	Fourier-transform infrared spectroscopy
UV–Vis	Ultraviolet–visible spectroscopy
OD	Optical density
ELISA	Enzyme-linked immunosorbent assay
PBS	Phosphate-buffered saline
STPP	Sodium tripolyphosphate
H&E	Hematoxylin and eosin
DPX	Dibutylphthalate Polystyrene Xylene (mounting medium)
IHC	Immunohistochemistry
HRP	Horseradish peroxidase
DAB	3,3′-diaminobenzidine
SDS-PAGE	Sodium dodecyl sulfate–polyacrylamide gel electrophoresis
TBST	Tris-buffered saline with Tween 20
BSA	Bovine serum albumin
ECL	Enhanced chemiluminescence
CAT	Catalase
SOD	Superoxide dismutase
NO	Nitric oxide
TNF-α	Tumor necrosis factor-alpha
IL-6	Interleukin-6
NGAL	Neutrophil gelatinase-associated lipocalin
Nrf2	Nuclear factor erythroid 2-related factor 2
BAX	BCL2 Associated X-protein
KIM-1	Kidney injury molecule-1
ANOVA	Analysis of variance
SD	Standard deviation
NS	Non-significant
