# Supplementary material for: Melittin Nanoparticles Mitigate Glyphosate-Induced Nephrotoxicity via Cytokine Modulation and Bax/Nrf2 Pathways
Source: Biomedicines. 2025 Oct 24;13(11):2607. doi: 10.3390/biomedicines13112607 (PMC12650702; doi:10.3390/biomedicines13112607)
Supplement: Supplementary file 1 [file biomedicines-13-02607-s001.zip › biomedicines-3898292-supplementary.pdf]

Supplementary Figures:

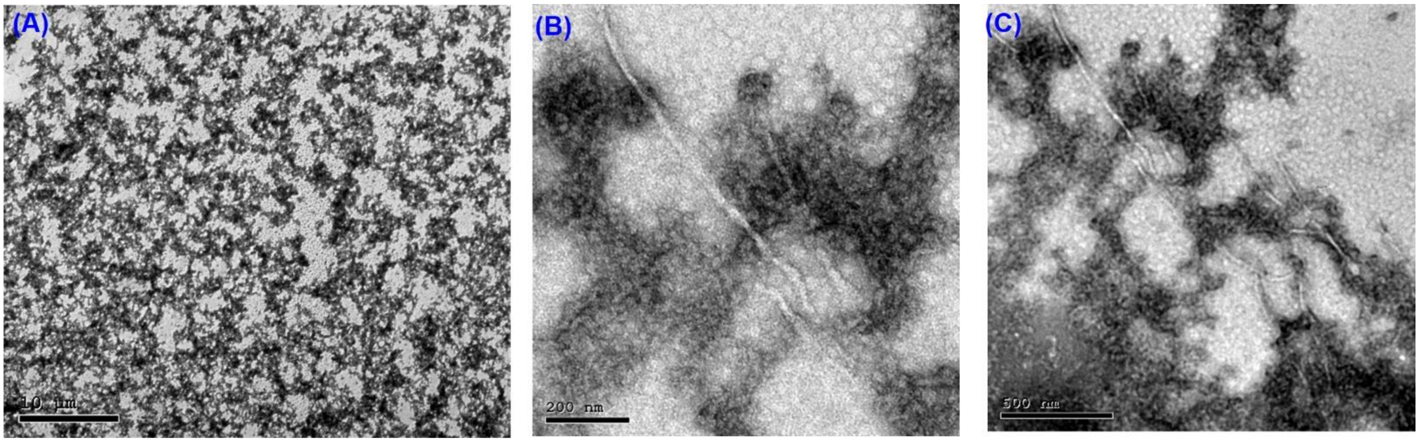

Figure S1. TEM micrographs of melittin-loaded chitosan–TPP nanoparticles (MEL-NPs) at different magnifications. (A) Low-magnification image (0.5 µm scale) showing a uniform distribution of MEL-NPs with spherical to slightly irregular morphology and minimal aggregation. **(B)** Medium-magnification image (200 nm scale) illustrating distinct particle boundaries and homogeneous size distribution within the nanometer range. **(C)** High-magnification image (500 nm scale) revealing the amorphous internal texture of the nanoparticles with smooth, well-defined surfaces. Overall, the TEM images confirm good dispersion, nanoscale size uniformity, and the absence of significant crystalline domains, consistent with amorphous chitosan-based nanostructures.
